# Use of the Airtraq® device for airway management in the prehospital setting – a retrospective study

**DOI:** 10.1186/1757-7241-22-10

**Published:** 2014-02-03

**Authors:** Mikael Gellerfors, Agneta Larsson, Christer H Svensén, Dan Gryth

**Affiliations:** 1 Department of Clinical Science and Education, Section of Anaesthesiology and Intensive Care, Karolinska Institutet, Stockholm South General Hospital, 118 83 Stockholm, Sweden; 2 Department of Physiology, Section of Anaesthesiology and Intensive care, Karolinska Institutet, 171 77 Stockholm, Sweden; 3 Falck Ambulance, Emergency Dispatch unit, Stockholm, Sweden

**Keywords:** Pre-hospital, Out-of-hospital, Prehospital emergency care (MeSH), Emergency medical services (MeSH), Helicopter emergency medical service, Critical care (MeSH), Airway management (MeSH), Endotracheal intubation (MeSH), Difficult endotracheal intubation, Complications (MeSH), Airtraq®, Patient safety

## Abstract

**Background:**

Difficulties with prehospital intubations have encouraged the development of indirect laryngoscopy techniques, facilitating laryngeal visualization. Airtraq® is a relatively new single-use indirect laryngoscope. The Airtraq® has been evaluated in several prehospital mannequin intubation trials. However, prehospital clinical experience with the device is limited.

**Methods:**

A retrospective medical chart review was performed for patients who underwent prehospital endotracheal intubation in the Stockholm County between January 2008 and December 2012. Both anaesthesiologists and nurse anaesthetists performed prehospital intubations during the study period. All Airtraq® intubations during this period were included in the analysis. The objective was to estimate the success rate of Airtraq® used in a prehospital setting.

**Results:**

During the 5-year period (January 2008- December 2012), 2453 tracheal intubations were performed. Airtraq® was used in 28 cases (1%). The overall Airtraq® intubation success rate was 68%. Among patients with anticipated or unexpected difficult airway (23/28) the Airtraq® success rate was 61% (14/23). Among patients who underwent drug facilitated or rapid-sequence intubation protocols 4/5 (80%) were successfully intubated with Airtraq®.

**Conclusion:**

In conclusion, this retrospective study showed a higher Airtraq® success rate than previous prospective prehospital trials. However, compared to other prehospital direct and indirect intubation methods the Airtraq success rate is low. Further clinical trials are necessary to evaluate the role of Airtraq® in the prehospital airway management.

## Introduction

Prehospital settings often present airway challenges [1]. Endotracheal intubation (ETI) is the ideal technique to secure an airway [2-5]. However, even if emergency medical personnel are adequately trained, the rate of complications is still high and failures are associated with morbidity and mortality [6-8]. Aggravating conditions such as non-ideal positioning and disturbing weather conditions must be taken under consideration. Difficulties with prehospital intubations have encouraged the development of several indirect laryngoscopy techniques facilitating laryngeal visualization [9]. The key feature of these indirect techniques is to enable visualization of the vocal cords without the need for aligning the oral, pharyngeal and tracheal axis. Indirect laryngoscopy may also be useful when the larynx is anteriorly located making regular intubation more difficult [5]. However, blood and mucus in the pharynx can considerably reduce the visibility and complicate indirect laryngoscopy [10]. One recent airway device is the Airtraq®(Prodol Meditec, Vizcaya, Spain). It was introduced in 2008 in the EMS helicopter and the emergency dispatch vehicle systems.

**Figure 1:**
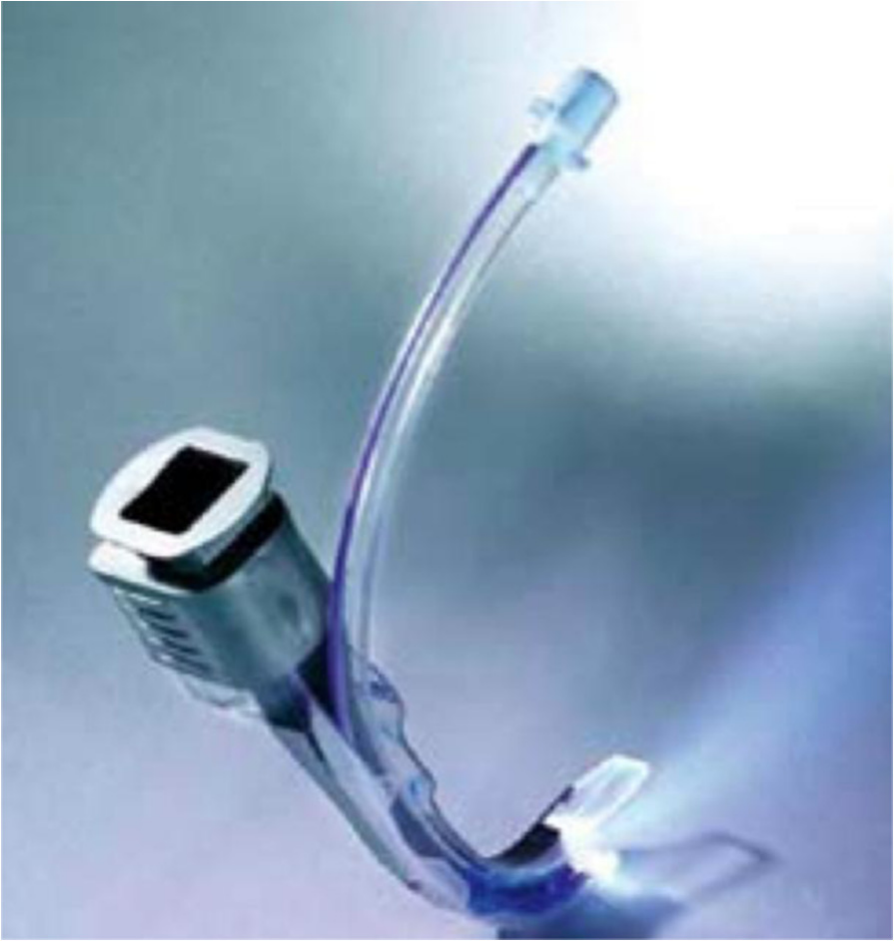
Airtraq®.

The Airtraq® is a battery powered indirect laryngoscope which has become popular in many EMS organizations. The Airtraq® has an optical channel with a distal lens that enables the user to visualize the glottis and a side channel where the tube is inserted (Figure 1). In several in-hospital studies, the Airtraq® has been shown to provide good endotracheal intubation conditions particularly when facing difficult airways [11-13]. The Airtraq® has also been evaluated in several prehospital mannequin intubation trials [14-16]. However, prehospital clinical experience with this device is limited [17,18]. In contrast to in-hospital trials, prehospital endotracheal intubation using the Airtraq® has been less successful when compared to direct laryngoscopy [18]. There are no data describing the prehospital efficiency of an Airtraq® device used as back up alternative when direct laryngoscopy has failed.

The objective of this study is to describe the Airtraq® efficiency when used as an alternative airway device in a prehospital setting. Furthermore in subgroup analysis we aim to investigate the Airtraq effectiveness in anticipated or unexpected difficult airway.

## Methods

### Study design

A retrospective medical chart review was performed for patients who underwent prehospital endotracheal Airtraq® intubation in the Stockholm County between January 2008 and December 2012.

### Setting

Stockholm County covers an urban area of approximately 6519 km^2^ with a population of 2.14 million inhabitants. The overall population density is 328 inhabitants pr. km^2^. Stockholm County has a two-tiered EMS system. The first tier consisted 2010 of 55 road ambulances staffed by a nurse with additional EMS training and a driver with basic emergency training. Some EMS nurses in Stockholm County may intubate cardiac arrests but are not trained to perform a drug-facilitated induction leading to rapid sequence intubation (RSI). Moreover, they do not use the Airtraq®, therefore these EMS providers could not be included in the study. The second tier consisted of four prehospital critical care teams staffed either with an anesthesiologist or a nurse anaesthetist. Three of these prehospital critical care teams are deployed by the emergency dispatch vehicle systems and the fourth team is working in the EMS helicopter. All prehospital critical care teams perform RSI as well as intubations during CPR. Many of them also work part-time in hospitals and are experienced in intubation procedures. They are also familiar with and regular users of indirect laryngoscopy techniques such as GlideScope, Storz C-MAC and/or McGrath depending on hospital.

During the study Airtraq® was used entirely at the discretion of the anesthesiologists or nurse anaesthetists. Only the second tier critical care teams were allowed to use the Airtraq® and there were no specific guidelines recommending the use of Airtraq®. Thus Airtraq® could be used both as a primary intubation device or when facing anticipated or unexpected difficult airways or when conventional direct laryngoscopy had failed. In cardiopulmonary resuscitation, Airtraq®-intubation was done without sedative or muscle relaxants. For non cardiac arrests RSI was the method of choice.

All prehospital critical care teams carry the same equipment for airway management. This includes equipment for bag-mask-ventilation (BMV), endotracheal tubes and standard laryngoscopes with Macintosh blades (Miller blades for infants and neonates), intubation stylets, Airtraq®, Gum-Elastic Bougies, laryngeal masks (LMAs), and equipment for establishing a surgical airway. All units are equipped with a capnography monitor and an automated ventilator.

### Participants

#### Inclusion criteria

All patients were an attempt to intubate with the Airtraq® had been done during the study period. The decision to use or to switch to an Airtraq® was completely at the discretion of the EMS anaesthesiologist or EMS anaesthesiology nurse on site.

#### Exclusion criteria

No patients intubated with the Airtraq were excluded from the study.

### Descriptive and exposure variables

Patient data, indications for intubation and the use of the Airtraq® were collected. The pre-hospital critical care teams were able to perform Airtraq-intubation with or without the assistance of drugs as RSI. EMS anaesthesiologist or EMS nurse anaesthetist could use a standard laryngoscope, the Airtraq®, LMA (Igel®) or establish a surgical airway. The intubation could be assisted with a standard intubation stylet and the gum-elastic bougie.

### Endpoints and outcome variables

Primary endpoint was Airtraq® intubation success rate. From the medical chart review, an Airtraq® intubation was defined as successful if the records showed that the responder had assessed the capnography and/or chest auscultation as satisfactory. The intubation was also considered successful if no information contradicted a successful intubation, such as no post Airtraq® intubation mask ventilation, laryngeal mask use or further direct laryngoscopy attempts. The latter additional definition of successful intubation is due to the retrospective nature of the study, with medical records often lacking information on auscultation and/or capnographic verification of the endotracheal tube. The prehospital instructions for intubation clearly state that capnography is recommended to verify that the tube is in a correct position. Failed intubations are all other Airtraq intubations not defined as successful.

Secondary endpoint was Airtraq® intubation success rate in anticipated or unexpected difficult airway. The anticipated or unexpected difficult airway was defined as failed laryngoscopy, failed LMA, foreign object in airway and/or trauma with the need for rigid collar/manual in-line stabilization (MILS).

### Data sources and data collection

The prehospital electronic medical record system in Stockholm County, CAK-net, was used to retrieve patient data. All prehospital medical records were sought for intubation, laryngoscopy and Airtraq. The data was managed in a Microsoft Database (Microsoft Corp.). The investigators MG and AL performed all data handling.

### Bias

All Airtraq® intubations are included in the retrospective analysis, reducing the risk for selection bias.

### Study size

This being a descriptive study, power calculation was not done.

### Statistical methods

The data was analyzed in the statistical program Excel (Microsoft Corp.)

### Ethics

The Regional Ethical Committee of the Stockholm County reviewed the study, and decided (No 2012/1668-31/4) that the study could be considered as part of quality control and thus did not need Ethical Approval.

## Results

**Figure 2:**
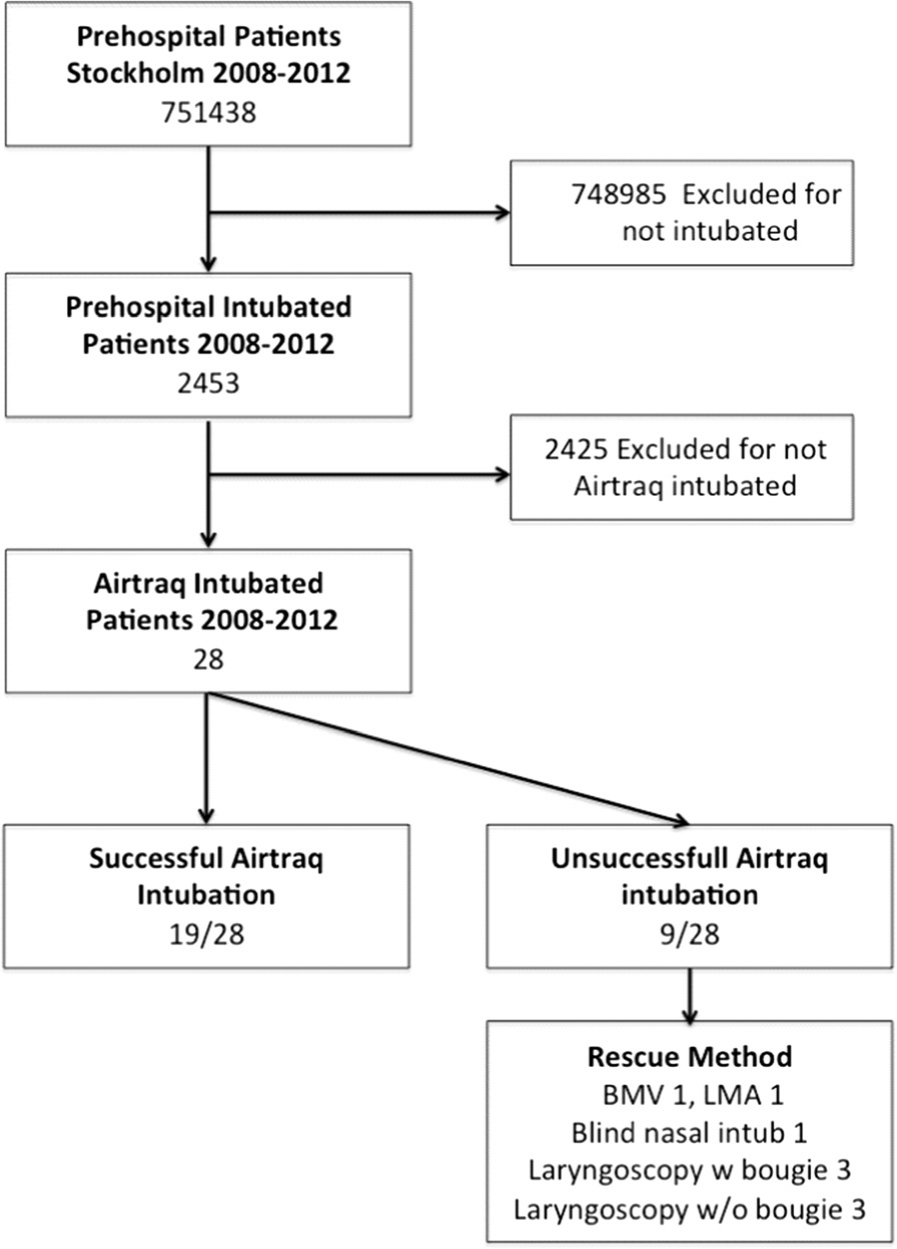
Airtraq® intubation flowchart.

During 2008-2012 there were 751438 patients admitted to the prehospital system (Figure 2). Of these, 2453 were intubated. The Airtraq® was used in 28 of these cases (1%). The majority of these patients were male (21/28, 75%). A total of 23/28 patients suffered from cardiac arrest. Five patients were intubated due to trauma and one due to respiratory insufficiency. The patient characteristics are shown in Table 1.

**Table 1:** Demographic data and indication for prehospital Airtraq intubation

**Demographic data**	**No patients (%)**
Average age (years)	52,4
Children <16 (n)	2
Gender (Female/Male)	7/21 (25/75)
Year 2008-2012	28
Indication for intubation	
Cardiac arrest	23/28 (82,1)
Foreign body	3/28 (10,7)
Hanging	2/28 (7,1)
Drowning	1/28 (3,6)
Trauma	5/28 (17,9)
Respiratory insufficiency	1/28 (3,6)
Drug facilitated intubation with suxamethonium.	5/28 (17,9)
Anticipated or unexpected difficult airway	23/28 (82,1)
Failed conventional laryngoscopy intubation	11/28 (39,3)
Failed laryngeal mask	2/28 (7,1%)
Intubated by Doctor/Nurse	14/14 (50/50)
Air/Ground rescue	3/25 (10,7/89,3)

Of the 28 cases where the Airtraq was used, 19 were considered successful (68%) (Table 2). The Airtraq® was used because of anticipated or unexpected difficult airways in 23 of the 28 (82%) intubations. Among these patients the Airtraq® intubation was considered successful in 14/23 cases (61%).

**Table 2:** Airtraq® intubation successrates

**Airtraq® efficacy**	**Successrate N (%)**
Children <16 (n)	1/2 (50)
Overall successrate	19/28 (67,9)
Cardiac arrest	15/23 (65,2)
Foreign body	2/3 (66,7)
Hanging	0/2 (0)
Drowning	1/1 (100)
Trauma	5/5 (100)
Drug facilitated intubation with suxamethonium	4/5 (80)
Anticipated or unexpected difficult airway	14/23 (60,8)
Prior failed conventional laryngoscopy intubation	4/11 (36,4)
Failed laryngeal mask (LMA)	2/2 (100)
Failed convent. laryngoscopy intubation or LMA	6/13 (46,2)

In 13 patients where the providers experienced previously failed airway management using conventional laryngoscopy or laryngeal mask, 6/13 (46%) were successfully intubated with Airtraq®.

Five patients required drug facilitated Airtraq® intubation with suxamethonium as a relaxant. Of these five patients four (80%) were successfully intubated with Airtraq®. Five patients were Airtraq® intubated after a trauma. In all cases the trauma was severe, requiring MILS. All (5/5, 100%) of these patients were successfully intubated with Airtraq®.

No fatalities were caused by failed airway management.

## Discussion

According to the updated American Society of Anesthesiologists Practice Guideline for Management of Difficult Airway 2013, video-assisted laryngoscopy may be considered as an initial approach in suspected difficult airway or when conventional ETI has been unsuccessful [19]. The objective of this medical chart review was to describe the use of an Airtraq® device as a facilitator for prehospital airway management. The main finding was that over a five-year period there were few cases where this device was used. The Airtraq 68% success rate was numerically higher in this retrospective study compared to previous prehospital prospective Airtraq® studies [17,18], despite the device often being used for an anticipated or unexpected difficult airway.

Prehospital airway studies are usually difficult to interpret since there are differences in the study design, definitions as well as in the recording and reporting of data. Furthermore there are large variations in the EMS systems involved including the EMS staffing. In this prehospital study 50% of the Airtraq intubations were performed by an anesthesiologist and 50% by an anesthesiology nurse. In the literature is generally reported that the airway management performance of physician-staffed EMS/HEMS [2,20-28] seems to be of a higher standard compared with that of paramedic-based systems [4,25]. However, the risks and complications of airway management in physician-staffed pre-hospital systems appears to be significant [25].

Several surveys show that there are still deficiencies in the availability of equipment for advanced airway management in ambulances and helicopters across Europe [29-31]. As a result, prehospital treatment of the emergency patient according to current guidelines [19,32] may not be possible in many areas. In the prehospital setting, EMS personnel are often faced with difficulties of endotracheal intubation, such as facial trauma, pharyngeal obstruction or limited access to the airway. The prehospital setting also presents less optimal conditions as regards to positioning, light and assistance [3,8,33]. Large variations in prehospital direct laryngoscopy total intubation success rates have been reported, ranging from 80% up to > 99% [12,34,35]. To improve visualisation of the airway and increase overall intubation success rates, the use of devices that enable indirect laryngoscopy, mainly by video sights, has become more frequent. The Glidesope Ranger video laryngoscope demonstrated a 97% success rate in 315 patients undergoing out-of-hospital intubation [36].

Although video laryngoscopes offer better visualisation of the glottis, a good laryngeal view does not imply easy or successful tracheal tube insertion [37,38]. All video laryngoscopes without an integrated guide channel for the endotracheal tube could face the challenge of advancing the tube into the trachea. The tip of the tracheal tube must pass through a critical angle to enter the larynx and has a significant risk of getting stuck on the anterior tracheal wall [39].

The Airtraq® has been shown to have a rapid learning curve for non trained personnel acquiring intubation skills [40,41]. After five minutes of Airtraq® training, a first time success rate of 79% have been reported for volunteer EMS personnel and experienced laryngoscopists achieved up to 84% success rate at first attempt in a difficult airway model [14,42]. In the current trial setting the majority of Swedish EMS personnel work at least part time in an anaesthesiology department and are familiar with indirect video laryngoscopes.

Most studies concerning video laryngoscopes and Airtraq® intubations have been performed on mannequins, simulating prehospital conditions. In a recent critical-care flight nurse and paramedic staffed HEMS prospective observational pilot trial (n = 50) Airtraq® was used as a first-line device for all intubations, resulting in a success rate of 62% [17]. In our present retrospective study Airtraq® permitted successful intubation in 68% of patients outside clinical trial setting. This finding is even more remarkable since 82% of patients presented with anticipated or unexpected difficult airway.

The results of our retrospective survey of the use of Airtraq® in Sweden 2008-2012 contradict the findings in the only prehospital prospective randomized Airtraq trial [18]. In the study by Trimmel et al the Airtraq, used by anaesthetists and EMS physicians rendered lower success rates (47% vs. 99%; p <0.001) when compared with direct laryngoscopy.

Speculating in the discrepancy between the 68% success rate when Airtraq® was used in the Swedish prehospital setting and the 47% success rate when used in the Austrian prospective study, some differences should be emphasized. Firstly, there are obvious important study design differences with a protocol only allowing two Airtraq® attempts in the Austrian trial. Secondly, the definition of successful intubation varies between the studies. In the retrospective study capnograph was not always used so all patients who did not have a medical record stating failed intubation or use of a rescue method was considered successful intubated. In the prospective study by Trimmel et al successful intubation was always confirmed with capnograph. Thirdly, 82% of the patients in our study had cardiac arrest whilst only 48% in the study by Trimmel. Patients with cardiac arrest lack protective airway reflexes, which may have made the Airtraq intubation easier in our study. Fourth, all EMS personnel in our study had an anaesthesia speciality education, which was not the case in the Austrian trial. It has been proposed that the Airtraq® is not taught to an acceptable performance level for the prehospital setting by the use of a manikin for training.

In trauma the cervical spine must be immobilised by a rigid collar or MILS, until the cervical spine injury has been excluded. The limited jaw opening and neck extension results in a Cormack and Lehane grade 3 or 4 in 64% of these cases [43]. In our chart review there was a prehospital Airtraq® intubation success rate of 100% in patients suffering from severe trauma.

There are several important limitations of this study. It is a very small number of cases retrieved from a medical chart review from one single center. The decision which airway device to use is not in accordance with a pre determined protocol. In our study this decision is subjectively made by the anaesthesiologists and nurse anaesthetists at the scene. Further, data extraction was performed by two of the authors, who were not blinded to the study objective. The majority of medical patient records were completed immediately after the procedure, however the accuracy of the information is still subject to self-report bias.

## Conclusion

A medical chart review from a prehospital setting in the Stockholm County showed a higher Airtraq® success rate than previous prospective trials. This could be due to differences in study design, patient characteristics and EMS provider level of anaesthesiology training. Compared to other prehospital studies of both indirect and direct laryngoscopy the Airtraq success rate is low. Randomised prehospital studies on Airtraq versus other indirect video laryngoscopes are needed to clarify the role of Airtraq in the prehospital setting.

## Competing interests

The authors declare that they have no competing interests.

## Authors’ contributions

MG, AL, CS, DG participated in the initiative of the research project and developed the study protocol. MG and AL performed the data collection and analysis. All authors contributed substantially in the interpretation of the findings and writing the manuscript. All authors read and approved the final manuscript.
